# Burnout and related factors in mothers of preschool children

**DOI:** 10.1007/s00737-025-01596-9

**Published:** 2025-05-31

**Authors:** Feyza Yılmaz, Pelin Göksel

**Affiliations:** 1 Samsun Mental Health Disorder Hospital, Samsun, Turkey; 2 Ondokuz Mayıs University, Adult Psychiatry, Samsun, Turkey

**Keywords:** Mother, Burnout, Spousal support, Parental stress

## Abstract

**Purpose:**

Being a parent, in addition to its rewarding aspects, also involves significant psychological and physical challenges. When the balance between caregiving burden and support resources is disrupted, it becomes almost inevitable for mothers to experience burnout. We designed our study to investigate the factors related to burnout complaints in mothers of preschool children.

**Methods:**

The sample of our study consisted of 80 mothers who presented to psychiatry clinics with burnout complaints and 80 healthy controls. Data collection tools included the Maslach Burnout Inventory, the Parenting Stress Index, and the Spousal Support Scale. Scale scores were compared between the groups. The mediating role of the Spousal Support Scale (SSS) in the relationship between the Parenting Stress Index (PSI) and the Maslach Burnout Inventory (MBI) was tested using path analysis.

**Results:**

No statistically significant age difference was found between the two groups (*p* > 0.05). The number of children among participants in the burnout group was higher (*p* = 0.018).In the burnout group, a positive and statistically significant relationship was found between the total scores of the Maslach Burnout Inventory (MBI) and the Parenting Stress Index (PSI) (r: 0.664; p: 0.000), and a negative and statistically significant relationship with the total score of the Spousal Support Scale (SSS) (r: -0.409; p: 0.000).The indirect effect of the Parenting Stress Index (PSI) on the Maslach Burnout Inventory (MBI) through the Spousal Support Scale (SSS) was found to be statistically significant ( $\:\beta\:$ = -0.3294, *p* < 0.001).

**Conclusion:**

Parental stress is associated with the level of burnout in mothers. However, spousal support may weaken the relationship between parental stress and burnout. Further research on the relationship between spousal support and burnout, as well as awareness projects related to spousal support, is needed.

## Highlights

Mothers presenting with burnout complaints had higher parental stress and lower spousal support.

Mothers’ burnout levels were positively correlated with parental stress and negatively correlated with spousal support.

Spousal support played a significant role in the effect of parental stress on burnout.

## Introduction

While children are considered a source of happiness for parents, their care also requires taking on a significant responsibility (You et al. 2019). In Turkey, as in many cultures, mothers are primarily responsible for child care. This situation brings challenges for working women in balancing home and work life, while for non-working women, it results in social isolation and the burden of taking on all household responsibilities (Demir 2018; Demircioğlu 2019). Additionally, women often become parents without adequate support and proper preparation, which can leave them with insufficient physical and mental resources to cope with the stress and caregiving burden that comes with parenthood (Verjus and Boisson 2005). All these challenges have been exacerbated by the demands and conditions of modern life in recent years, making “burnout in mothers” an important research topic (Eustache 2016; Fidan, 2016).

Burnout was first defined as a concept related to work life by Maslach (Maslach 1976), while parental burnout was introduced by Maslach and Jackson(Maslach and Jackson 1981), considering the similar challenges of work life and child care. Parental burnout arises from the imbalance between the demands of child care and the available resources (Mikolajczak and Roskam 2018). As a result, various factors such as the number of children a woman has, the ages of her children, the presence of a child with special care needs, work status, the adequacy of environmental and spousal support, coping attitudes, physical and mental health state, parental stress all influence parental burnout (Sabzi et al. 2023; Vigouroux and Scola 2018).

Adequate spousal and social support, as well as the ability to utilize adaptive coping methods, can be seen as protective factors against parental burnout (Çalışkan et al. 2021; Güler and ÇAPRİ 2019; Seo and Kim 2022). Moreover, the division of responsibilities between parents, along with the exchange of empathy and positive emotions, positively impacts a child’s emotional and cognitive development (Gottman and Notarius 2000) Considering that parenthood is a demanding and burdensome responsibility, it is expected that having a reliable and supportive partner, as well as being able to trust in that partner’s support, would alleviate the burden felt regarding parenting duties (Favez et al. 2019) However, as studies on marriage have shown, recurring significant conflicts, a lack of empathy, and an inability to appropriately divide responsibilities can strain partners’ emotional resources and lead to burnout (Gottman and Notarius 2000; Teubert and Pinquart 2010).

Another concept closely related to parental burnout is parental stress, which refers to the emotional strain experienced by individuals while raising children (Abidin 1983). A study conducted with a sample of 105 mothers whose children attend daycare demonstrated a significant correlation between burnout and parental stress among the participants (*r* = 0.62, *p* < 0.001) (Seo and Kim 2022). Another study conducted in 2018 included 304 mothers and concluded that parental stress significantly predicted all three subdimensions of burnout (emotional exhaustion, depersonalization, and personal accomplishment). (Lebert-Charron et al. 2018). In conclusion, parental stress acts as an additional strain within the burden/resource balance associated with burnout, tilting the scale towards burnout (Sánchez-Rodríguez et al. 2019).

**Fig. 1:**
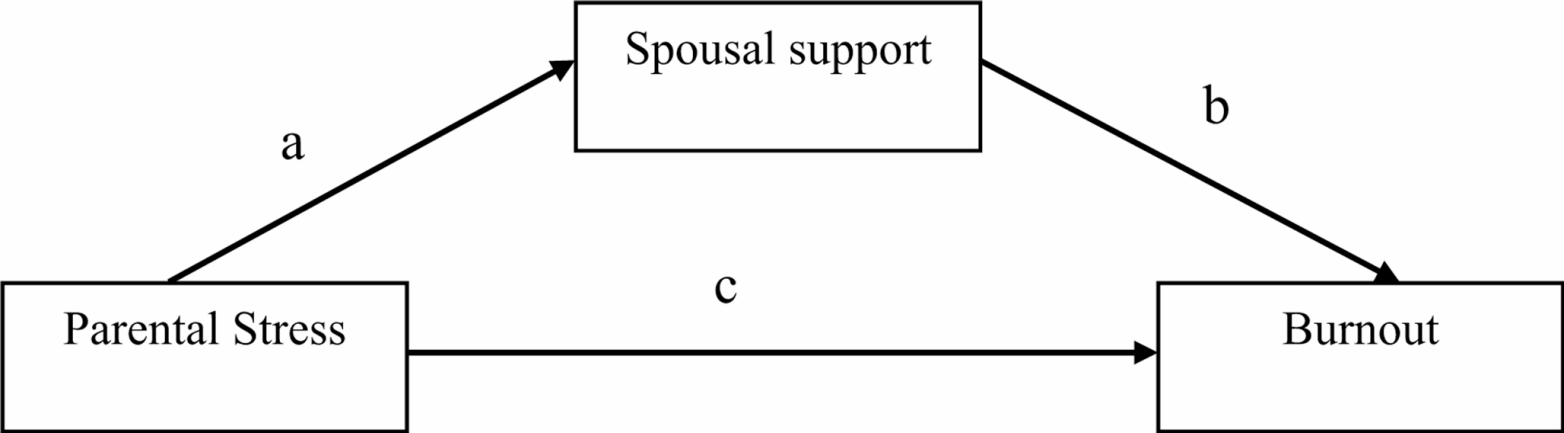
Path analysis model established between spousal support, parental stress, and burnout scales

Parental burnout continues to be an intriguing and important area of research in recent years. However, factors associated with parental burnout in women have not been sufficiently studied. In our sample, composed of women who presented to a psychiatry clinic with burnout complaints and have at least one preschool child, our aim is to investigate the relationship between parental burnout and sociodemographic characteristics, spousal support, and parental stress, as well as the mediating role of spousal support in the relationship between parental stress and burnout. The model illustrating the mediating role of spousal support is presented in Fig. 1.

## Methods

A review of the literature regarding the concepts of “mother” and “burnout” reveals that the most frequently reported complaints include feeling unable to care for children, believing that one is no longer a good mother, feeling guilty about parental roles, experiencing tension and irritability due to parenting responsibilities, and feeling drained or exhausted by child care(Brianda 2021; Paula et al. 2021; Séjourné et al. 2018). Based on this information, our study sample was composed of mothers who presented to psychiatry clinics with burnout and complaints closely related to the concept of burnout. The study included married women aged 18 and older who have at least one preschool child. Individuals with insufficient mental capacity, those experiencing a psychotic episode, and those with severe physical illnesses were excluded from participation in the study. Informed consent was obtained in writing from all participants.

Data were collected using personal information questionnaires between February 1, 2024, and June 15, 2024.

### Data collection tools

#### Maslach Burnout scale

The scale developed by Christina Maslach and Jackson consists of 22 questions and three subdimensions (Maslach and Jackson 1981). The reliability coefficients for the subscales were calculated as 0.89, 0.86, and 0.77, respectively. A validation study of the inventory was conducted on a sample of mothers by Pelsma et al. in 1989 (Pelsma et al. 1989). A validity and reliability study in Turkish was conducted by Ergin. The three-dimensional structure of the scale was maintained in the Turkish version as well. In the analyses conducted as part of the adaptation study, the Cronbach alpha internal consistency coefficients were calculated as 0.83 for the emotional exhaustion subscale, 0.72 for the personal accomplishment subscale, and 0.65 for the depersonalization subscale (Ergin 1992). A validity and reliability study of the scale on a sample of parents was conducted by Duygun and Sezgin. In this version, the scale items were grouped into two factors: “emotional exhaustion” and “personal accomplishment.“Additionally, the 15th item from the original form was removed because its factor loading was below 0.30 (Sezgin and Duygun 2003). In our study, the revised form of the Maslach Burnout Inventory for parents was used.

### Parental stress index

The scale developed by Abidin consists of three subdimensions: Parental Distress, Dysfunctional Parent-Child Interaction, and Child Difficulties (Abidin 1983). The scale consists of 36 items, with 12 items in each subdimension, and uses a 5-point Likert type format.

The adaptation to Turkish was carried out by Mert et al (Mert et al. 2008). The Cronbach alpha internal consistency coefficients were calculated as 0.81 for the Parental Distress subscale, 0.76 for the Dysfunctional Parent-Child Interaction subscale, and 0.78 for the Difficult Child subscale.

### Spousal support scale

The scale developed by Yıldırım consists of 27 items and four subdimensions (Yildirim 2004). In the internal consistency analysis conducted, the internal consistency coefficients for the subdimensions of emotional support, material assistance, informational support, appreciation support, and social interest support were calculated as 0.91, 0.87, 0.79, 0.68, respectively, with a total scale coefficient of 0.95.

### Ethical board approval

Our study was approved by the Amasya University Clinical Research Ethics Committee on January 17, 2024 (2023000160-1).

### Statistical analysis

Scale scores for normally distributed groups were compared using the T-test or One-Way ANOVA, while non-normally distributed groups were compared using the Mann-Whitney U or Kruskal-Wallis tests. The relationships between continuous variables were assessed using Pearson or Spearman correlation tests. In multiple group comparisons, Bonferroni correction was applied to prevent Type 1 errors. In the burnout group, categorical variables that showed differences between groups in terms of Maslach Burnout Inventory total scores, as well as continuous variables that showed a statistically significant correlation with the Maslach Burnout Inventory, were included in the linear regression analysis. The Maslach Burnout Inventory was considered the dependent variable. The mediating role of the Spousal Support Scale (SSS) in the relationship between the Parental Stress Index (PSI) and the Maslach Burnout Inventory (MBI) was examined using path analysis. In the established path analysis model, the standardized regression coefficients representing the relationships between variables, along with the confidence intervals for these coefficients, were estimated using the unweighted least squares method, as the assumption of multivariate normality was not met. The fit of the path analysis model to the data was assessed using the fit indices CFI, GFI, and RMSEA. In all statistical analyses, p-values less than 0.05 were considered significant.

## Results

When analyzing the study data, the average age of the burnout group was calculated as 31.7 ± 4.99, while the average age of the control group was 30.94 ± 4.73. No statistically significant age difference was found between the two groups (*p* > 0.05). The number of children held by participants in the burnout group was higher (*p* = 0.018). There was no significant difference in the presence of developmental disorders (such as autism, attention-deficit and hyperactivity disorder or intellectual disability) among the children (*p* = 0.789). While no significant difference in education level was found between the two groups, a higher proportion of participants in the control group were employed. Sociodemographic and clinical variables related to the burnout and control groups are summarized in Table 1.

**Table 1:** Comparison of sociodemographic variables between groups

Categorical Variables (Number (%))		Burnout Group	Control Group	*p*-value
Education Status	Primary Education	24 (15%)	17 (10,6%)	0,057
	High School	29 (18.1%)	21 (13,1%)	
	University	27 (16.9%)	42 (26.3%)	
Employment Status	Unemployed	59 (36.9%)	32 (20%)	< 0.001
	Employed	21 (13,1%)	48 (30%)	
Place of Residence	Village	13 (8.1%)	6 (3.8%)	0,16
	District	19 (11.9%)	26 (16.3%)	
	City	48 (30%)	48 (30%)	
Income Status	< 10k	11 (6.9%)	3 (1.9%)	0.021
	10–50 thousand	55 (34.4%)	52 (32.5%)	
	> 50,000	14 (8.8%)	25 (15.6%)	
Chronic Disease	None	59 (36.9%)	64 (40%)	0,348
	Yes	21 (13,1%)	16 (10%)	
Psychiatric Disease	None	57 (35.6%)	74 (46.3%)	< 0.001
	Yes	23 (14.4%)	6 (3.8%)	
Disease in Child	None	73 (45.6%)	72 (45%)	0,786
	Yes	7 (4.4%)	8 (5%)	
Care Support	None	51 (31.9%)	33 (20.6%)	0.004
	Yes	29 (18.1%)	47 (29.4%)	
If Caregiver Support is Present, Who?	Babysitter	3 (4.1%)	5 (6.7%)	0.983
	Mother/Relative	25 (33.8%)	41 (55.4%)	
**Continuous variables (Mean ± SD)**		**Burnout Group**	**Control Group**	**p-value**
Age		31.69 ± 4.98	30.94 ± 4.73	0,331
Number of children		1.89 ± 0.87	1.59 ± 0.7	0.018

SD: standard deviation

In the burnout group, the total and subscale scores of the Maslach Burnout Inventory (MBI) and the total and subscale scores of the Parenting Stress Index (PSI) were higher compared to the control group, while the total and subscale scores of the Social Support Index (SSI) were lower compared to the control group. All observed differences are statistically significant. Comparisons of total and subscale scores of the scales are presented in Table 2.

**Table 2:** Comparison of scale scores between the burnout and control groups

	Control Group		Burnout Group		*P*-Value
	Mean ± SD	Median	Mean ± SD	Median	
MBI-total^1^	35,575 ± 12,580	32,50	48.275 ± 16.432	45,00	< 0.01
MBI- emotional burnout^2^	18.4875 ± 7.023	18,00	24.2 ± 9.224	23,00	< 0.01
MBI-depersonalization^1^	7.3 ± 3.070	6,00	10.7375 ± 4.765	10,50	< 0.01
MBI- personal success^1^	9.85 ± 5.289	8.50	13.2875 ± 6.524	13,00	< 0.01
PSI- total^2^	64.87 ± 21.66	59,00	90.6375 ± 27.253	87,50	< 0.01
PSI-parental distress^2^	24.86 ± 9.85	24,00	34.625 ± 10.848	35,00	< 0.01
PSI-Parent-Child Dysfunctional Interaction^1^	18.26 ± 6.87	16,50	25.15 ± 11.019	23,00	< 0.01
PSI-Difficult Child^2^	22.0375 ± 8.276	21,00	30.3125 ± 11.156	31,00	< 0.01
SSS-total^2^	65.575 ± 10.128	68,00	55.425 ± 12.362	58,00	< 0.01
SSS-emotional support^2^	23.2125 ± 4.121	24,00	19.5625 ± 5.286	20,00	< 0.01
SSS-financial support^2^	16.925 ± 2.661	17.50	14.8 ± 3.354	16,00	< 0.01
SSS- appreciation support^2^	17.7375 ± 2.967	18,00	15.2375 ± 3.490	16,00	< 0.01
SSS- social support^2^	7.7125 ± 1.544	8,00	6.05 ± 1.961	6,00	< 0.01

^1^ Mann Whitney U, ^2^T test

MBI: Maslach Burnout Inventory, PSI: Parent Stress Index, SSS: Spousal Support Scale

In the comparisons based on sociodemographic variables within the burnout group, it was found that the total score of the Maslach Burnout Scale had a positive correlation with age and the number of children. No significant relationship was found between the total score of the Maslach Burnout Scale and other sociodemographic variables (*p* > 0.05). In the subscale comparisons, it was found that the Maslach Depersonalization subscale score was higher in those who did not receive caregiving support (t: 2.836; p: 0.006), and the Maslach Emotional Exhaustion subscale score was higher in those with a developmental disorder in their child (U: 121.5; p: 0.022). After applying the Bonferroni correction, the difference found in the Maslach Depersonalization subscale maintained its statistical significance, while the difference in the Maslach Emotional Exhaustion subscale lost its statistical significance. The comparison of the Maslach Burnout Scale with sociodemographic data is summarized in Table 3.

**Table 3:** Comparison of MBI scores with sociodemographic data in the burnout group

	MBI total		
Continuous variables (Mean ± SD)	Mean ± SD	Median	*p*-value
Age	31,69 ± 4,98	r: 0.221, p: 0.048	31,69 ± 4,98
Number of children	1.89 ± 0.87	r: 0.387, p: <0,001	1.89 ± 0.87

SD: standard deviation

In the burnout group, the total score of the Maslach Burnout Inventory is 48.28 ± 16.43, the total score of the Partner Support Scale is 55.43 ± 12.36, and the total score of the Parent Stress Index is 90.64 ± 27.25. In the correlation analysis conducted, a positive and statistically significant relationship was found between the total scores of the Parent Stress Index (r: 0.664; p: 0.000) and a negative and statistically significant relationship with the total score of the Partner Support Scale (r: -0.409; p: 0.000). The data related to the correlation analysis are presented in Table 4.

**Table 4:** Correlation analysis for burnout group

		PSI total	PSI-parental distress	PSI -Parent-Child Dysfunctional Interaction	PSI-Difficult Child	SSS-total	SSS-emotional support	SSS-financial support	SSS- appreciation support	SSS- social support
MBI -Total	r	,664^**^	,618^**^	,626^**^	,390^**^	-,409^**^	-,392^**^	-,267^*^	-,337^**^	-,411^**^
	p	0,000	0,000	0,000	0,000	0,000	0,000	0,017	0.002	0,000
MBI- emotional burnout	r	,635^**^	,623^**^	,537^**^	,392^**^	-,381^**^	-,350^**^	-,270^*^	-,329^**^	-,402^**^
	p	0,000	0,000	0,000	0,000	0,000	0.001	0,015	0,003	0,000
MBI-depersonalization	r	,624^**^	,559^**^	,582^**^	,416^**^	-,339^**^	-,290^**^	-,220^*^	-,301^**^	-,372^**^
	p	0,000	0,000	0,000	0,000	0.002	0.009	0,050	0,007	0.001
MBI- personal success	r	,321^**^	,258^*^	,392^**^	0,137	-,250^*^	-,284^*^	-0,133	-0,175	-0,199
	p	0.004	0.021	0,000	0.225	0.025	0,011	0,241	0.120	0.077

MBI: Maslach Burnout Inventory, PSI: Parent Stres Index, SSS: Spousal Support Scale, *The correlation is significant at the 0,01 level, **The correlation is significant at the 0,05 level

After identifying the variables associated with the Maslach Burnout Inventory in the burnout group, these variables were subjected to linear regression analysis, with the Maslach Burnout Inventory considered as the dependent variable. The analysis revealed that the number of children (B: 4.53; p: 0.027) and the total score of the Parent Stress Index (B: 0.326; p: 0.000) maintained their significance, while the significance of the other variables was lost. According to the analysis, the model explains 45% of the variance in the dependent variable, the Maslach Burnout Inventory. The findings of the linear regression analysis are presented in Table 5.

**Table 5:** Linear regression analysis of MBI, number of children, and PSI

	Non-standardized Coefficient		Standardized Coefficient	t	*P*-Value	95.0% Confidence interval for B	
	B	Standard Error	Beta			Lower limit	Upper limit
Number of Children	4.533	2,013	0,240	2.252	0,027	0,518	8,547
PSI	0,326	0.063	0.541	5.187	0,000	0,201	0.452

PSI: parent Stres Index

The results of the path analysis model testing whether the SSS has a mediating role in the relationship between PSI and MBI are presented below.

When examining the results in Table 6, the indirect effect of the Parenting Stress Index (PSI) on the Maslach Burnout Inventory (MBI) through the Spousal Support Scale (SSS) was also found to be negative and statistically significant ($\:\beta\:$ = -0.3294, *p* < 0.001). In this case, it has been observed that the Spousal Support Scale (SSS) has a mediating effect in the relationship between the Parenting Stress Index (PSI) and the Maslach Burnout Inventory (MBI). The goodness-of-fit indices indicating whether the path analysis model investigating the mediating effect of the Spousal Support Scale (SSS) is compatible with the data are as follows: CFI: 1.000 (> 0.95 indicates very good fit), GFI: 1.000 ( > 0.95 indicates very good fit), and RMSEA: 0.000 ( < 0.05 indicates very good fit), which are considered acceptable limits. Accordingly, it can be said that the established path analysis model fits the data very well.

**Table 6:** The results of the path analysis model established between the SSS, PSI, and MBI

	Standardized $\:\varvec{\beta\:}$	Confidence Interval	*p*-value
Direct Impact			
SSS→ MBI	0.3409	0,3404; 0,3414	< 0.001
PSI→ SSS	-0.2471	-0.2473; -0.2469	< 0.001
SSS→ MBI	-0.2416	-0.2432; -0.2399	< 0.001
Indirect effect			
PSI→ SSS→ MBI	-0.3294	-0.3299; -0.3290	< 0.001

CFI: 1,000, GFI: 1,000, RMSEA: 0,000

SSS: Spousal Support Scale, PSI: Parenting Stress Index, MBI: Maslach Burnout Inventory

## Discussion

In our study investigating the factors associated with the burnout levels of women with preschool children, we found that the parental stress levels of women who presented to the psychiatry outpatient clinic with burnout complaints were higher and the support they received from their spouses was lower compared to the control group. The burnout levels of participants in the burnout group were positively correlated with age, number of children, and Parenting Stress Index (PSI) scores, while they were negatively correlated with Spousal Support Scale (SSS) scores. In the linear regression analysis conducted, only the number of children and the Parenting Stress Index (PSI) retained their significance.

When the literature is reviewed, inconsistent results are found regarding the relationship between parental age, number of children, and burnout levels. A study examining the factors related to parental burnout during the Covid-19 quarantine found that parents with children under the age of four and younger parents showed more signs of burnout, while no significant relationship was found between the number of children and burnout symptoms (Woine et al. 2022). In the study conducted by Le Vigourous et al., the number of children and parental age were not found to be associated with parental burnout and its subdimensions (Le Vigouroux et al. 2022). A study addressing the impact of demographic factors on parental burnout found that being a younger parent and having a higher number of children in the family increased burnout, but the average age of parents was not related to burnout (Vigouroux and Scola 2018). However, previous studies have shown that mothers experience more symptoms of burnout if their children have physical or mental disabilities (Gérain and Zech 2018tük et al., 2021). In our study, the presence of any developmental disorder in the child was not found to be associated with burnout levels. We believe that this may be due to the relatively small sample size of our study.

In our study, a significant relationship was found between parental stress and burnout. Additionally, almost all of the correlations between the subscales of the Maslach Burnout Inventory and the subscales of the Parental Stress Index (PSI) were found to be significant. A study conducted by Aktu with Turkish parents found that parental stress positively predicted parental burnout, and that parental self-efficacy played a mediating role in this relationship (Aktu 2024) A study conducted with mothers of children with autism spectrum disorder in China concluded that parental stress is a risk factor for parental burnout, and that resilience may be a potential underlying mechanism for this relationship (Liu et al. 2024). A study conducted during the Covid-19 pandemic found that parental burnout was positively associated with parental stress, while perceived social support was negatively related to both parental burnout and parental stress (Vaydich and Cheung 2023). In conclusion, there is a substantial body of literature focusing on the relationship between parental stress and burnout, and the findings are generally consistent, indicating that our study results support the existing literature.

Our study found that parental burnout is negatively associated with social support. Moreover, almost all of the correlations between the subscales of the MBI and the subscales of the PSI were found to be significant. The literature on the relationship between social support and parental burnout is quite limited. According to the results of a study examining the relationship between co-parenting and burnout in dual-parent families in Switzerland, positive subdimensions such as support, closeness, and agreement showed a negative correlation with burnout. Additionally, the study concluded that having a higher number of children and having younger children also increased burnout (Favez et al. 2023). In a large-sample study conducted in Japan, being a single parent and having less spousal support were found to be predictors of burnout (Furutani et al. 2020). Another study found that perceived spousal support reduced postpartum depression symptoms (Adil et al. 2021). In light of the current information, it can be interpreted that there is a relationship between spousal support and burnout symptoms in mothers; however, the factors related to this relationship have not been adequately researched.

The results of our study indicate that parental stress has a direct and positive effect on burnout ($\:\beta\:$= 0.3409, *p* < 0.001). Moreover, it was found that spousal support plays a mediating role in the relationship between parental stress and burnout. When the literature on parental stress is reviewed, it is generally observed that it focuses on factors related to an individual’s internal resources, such as coping styles, psychological well-being, and resilience (Lisanti 2018; Upadhyay and Parashar 2022). Several studies have shown that perceived social support reduces parental stress and improves burnout outcomes (Chen et al. 2022; Martinez and Turnage 2022) However, none of these studies specifically focused on spousal support. However, spousal support plays a significant role in a mother’s ability to cope with the physical and psychological burdens of child care and to combat feelings of burnout. Our study is the first to examine the role of spousal support in the relationship between parental stress and burnout.

### Limitations

Our study has some limitations. Firstly, our study’s burnout group was composed of participants who frequently presented with burnout complaints at a psychiatry outpatient clinic, and no diagnostic tools were used. When the findings were examined, it was seen that the burnout group participated in work life less than the control group, had a worse income status, had more psychiatric disease diagnoses and had worse care support. These differences were found to be statistically significant. However, none of these variables showed a significant relationship with MBI. Nevertheless, it is possible to reach more accurate results with a more homogeneous group in terms of sociodemographic characteristics. This situation constitutes a limitation for the study. On the other hand, there are many variables that could influence the relationship between parental stress, spousal support, and burnout (such as coping styles and working conditions). The insufficient assessment of these variables in our study also constitutes a limitation. We hope that future studies with larger sample sizes and investigations of different variables will provide enlightening findings on the topic.

## Conclusion

In summary, in our study examining the factors associated with burnout levels in women with preschool-aged children, we found that women presenting with burnout complaints at a psychiatry outpatient clinic had higher parental stress and lower spousal support compared to healthy controls. There was a negative mediating effect of spousal support in the relationship between parental stress and burnout. Our results highlight the need for awareness campaigns and social policies regarding the importance of spousal support.
